# Patterns of Diabetic Complications at Jimma University Specialized Hospital, Southwest Ethiopia

**DOI:** 10.4314/ejhs.v20i1.69424

**Published:** 2010-03

**Authors:** Dawit Worku, Leja Hamza, Kifle Woldemichael

**Affiliations:** 1 Department of Medicine, Health Science College, Adama University; 2 Department of Internal Medicine, College of Public Health and Medical Sciences, Jimma University; 3 Department of Epidemiology, College of Public Health and Medical Sciences, Jimma University

**Keywords:** Diabetes mellitus, chronic complications, Southwest Ethiopia

## Abstract

**Background:**

Diabetes Mellitus is common metabolic disease worldwide. Its complications in the Ethiopian care setup has not been well documented. The objective of this study was to assess the pattern and distribution of diabetic complications among patients having follow-up at Jimma University specialized Hospital diabetic clinic.

**Methods:**

A cross sectional study based on record review of 305 patients, selected using systematic sampling with replacement was carried out in October 2008. The data were analyzed using SPSS for Windows version 13.0.

**Results:**

Larger proportion, 189 (62.0%), of patients had type II diabetes and 163 (53.4%) of them were diabetic for less than 5 years. Seventy three of the 76 (96.1%) patients with type II diabetes mellitus had hypertension. Acute complications were observed in 93 (30.5%) of the patients of which Diabetic Ketoacidosis was documented in 66(71.0%).

Forty eight (45.7%) of patients had proteinuria, 90 (29.5%) had peripheral neuropathy, 13(6.8%) had impotence. Diabetic foot ulcer, skin and/or subcutaneous tissue infection, dental problems and tuberculosis were documented in 14(4.5%), 31(10.0%), 31(10.0%), and 17(5.6%) patients, respectively. Any of the chronic complications were not different by sex of the patient but age had statistically significant association with hypertension, visual disturbance and neuropathy (p< 0.05). Type of diabetes had statistically significant association with all the tested complications except infection (P<0.05) where most of the complications occurred in type II diabetics. Statistically significant association was observed between the duration of the diabetes and impotence and visual disturbances (p < 0.05).

**Conclusion:**

The majority of patients were type II diabetics. Acute complications were observed more commonly among type I diabetics and DKA was the commonest acute complication. The frequency of chronic complications was high. Increased occurrence of retinopathy, peripheral neuropathy, hypertension and nephropathy was observed with longer duration of illness. Impotence and diabetic nephropathy were more common in type II diabetics. The study showed that age, sex, type of diabetes mellitus and duration of diabetes were significantly associated with the development of diabetic complications.

## Introduction

Diabetes mellitus is the commonest of all metabolic diseases all over the world. The world wide prevalence of diabetes mellitus has increased dramatically over the past decades from an estimated 30 million cases in 1985 to 177 million in 2000. A recent estimate suggested that diabetes mellitus was the 5^th^ leading cause of death worldwide and is responsible for almost 3 million deaths annually (1).

Based on the current trend more than 360 million individuals will have diabetes by the year 2030. WHO estimates the number of cases of diabetics in Ethiopia to be about 800,000 in 2000 and projected that it would increase to about 1.8 million by the year 2030(2).

Diabetes mellitus leads to acute and chronic complications. The acute complications include diabetic ketoacidosis (DKA), hyperosmolar hyperglycemic state (HHS), and hypoglycemia during treatment and the chronic complications are neuropathy, nephropathy, retinopathy, ischemic heart disease, myocardial infarction, stroke, peripheral arterial disease, impotence and so on (1).

Among 849 consecutive Ethiopian diabetics at Yekatit 12 Hospital in 1984, 20.1% were Insulin Dependent Diabetes Mellitus (IDDM), 79.1% Non Insulin Dependent Diabetes Mellitus (NIDDM), 0.5% drug induced and 0.1% gestational diabetes mellitus (3).

Out of a total of 283 diabetic patients (112 types I and II) followed at Menilik II hospital in Addis Ababa, 106 (37.5%) had chronic complications (33% type I and 16.9% type II) with diabetic nephropathy showing significant difference between type I &II patients. Retinopathy and peripheral neuropathy were seen in 31.4% and 35.2%, respectively (4).

Studies done at Tikur Anbessa Hospital showed an overall prevalence of diabetic retinopathy 37.8 % (5) and impotence 48.7% (6) and infection 44% (7). Diabetes mellitus was also reported to be the leading cause of non-traumatic lower extremity amputation in the United States (1). Although diabetes mellitus is being recognized as one of the health problems, its complications in the Ethiopian care setup has not been well documented. Information on diabetic complications could help in tuning up the service delivery for diabetic patients. Therefore, the objective of this study was to assess the pattern and distribution of diabetic complications among patients having follow-up at Jimma University specialized Hospital's diabetic clinic.

## Methods and Materials

A cross-sectional study based on using records of 305 diabetic patients was conducted in October 2008 at JUSH's diabetic follow-up clinic, which is located in Jimma City, 352km Southwest of Addis Ababa. The hospital is a tertiary hospital and gives health service for more than 10, 000,000 people living in Southwest Ethiopia. There were about 1460 diabetic patients who have been following diabetic clinic since 2004. The weekly diabetic follow-up clinic gives service to 70–90 patients per day.

The sample size was determined with the following assumptions: an expected prevalence of complication 50%, margin of error 5%, at 95% confidence level with corrections for finite population which gave a sample size of 305. Using systematic sampling, every fifth card was selected starting from the lowest card number in ascending order. When the fifth card was missing, the next card was used. Data regarding patient characteristics, prevalence and pattern of complications of DM was retrieved from patients chart on a prepared format. The data collection process was supervised and the data were cross checked.

Data were analyzed using SPSS for window version 13.0. Association between different variables was tested for statistical significance. Result was presented in tables and graphs.

The proposal was endorsed by the ethical Review committee of Medical Sciences Faculty of Jimma University. Permission was obtained from the Hospital administration and the diabetic clinic staff before the start of data collection.

Operational definition

- Type II with secondary failure: Patient who was previously diagnosed to have type II DM and was taking oral anti-diabetic drugs but started on insulin due to failure of control of serum glucose level by the oral anti-diabetic drugs.
- Hypoglycemia: Blood glucose level <50mg/dl or symptoms of hypoglycemia which are recorded by the physician.

## Results

Of the 305 reviewed charts, 116 (38.0%) patients had type I diabetes, 153 (50.2%) type II and 36 (11.8%) type II diabetics with secondary failure. The mean (±SD) age of the patients was 44.4 (±15.6) years, and 192 (62.9 %) were males. The majority of patients, 163 (53.4%), had been diabetics for less than 5 years. Hypertension was present in 76 (24.9%) of the patients. Majority, 88 (75.8%), of type I diabetics were in the age group 15–34 while 78 (41.2%) of type II diabetics were in the age group more than 55. Of those diabetic patients with hypertension 73 (96.1%) were type II diabetics (*Table 1*).

**Table 1:** Distribution of Diabteic patients by sex, age, duration of illness and type of diabetes,, JUSH, October 2008.

Variables (n=305)		Frequency (n=305)	Percent
**Sex**			
	Male	192	63.0
	Female	113	37.0
**Age**			
	15–34	94	30.8
	35–44	54	17.7
	45–54	77	25.3
	≥55	80	26.2
**Type of DM**			
	Type I	116	38.0
	Type II	153	50.2
	Type II with 2^0^ failure	36	11.8
**Duration of diabetes (Year)**			
	<5	163	53.4
	5–9	103	33.8
	10–14	28	9.2
	15–19	6	2.0
	>20	5	1.6

**Fig 1:**
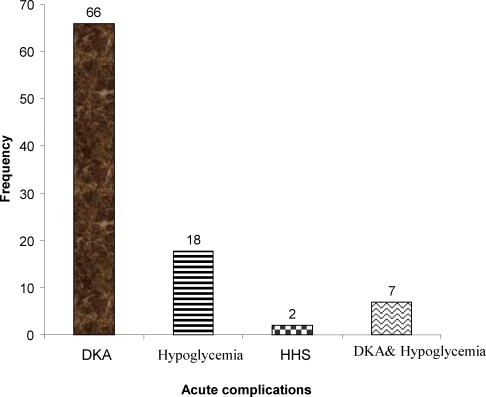
Acute diabetic complications, Jimma University specialized Hospital, October 2008. (HHS: Hyperosmolar Hyperglycemic State, DKA: Diabetic ketoacidosis)

Acute diabetic complication was documented in 93 (30.5%) patients where DKA was the commonest accounting for 71% followed by hypoglycemia, 19.4%. Two patients had HHS and both were type II diabetics and other seven patients had both DKA and hypoglycemia (*Fig 1*).

Among the 305 patients in the study, 160(52.5%) had one or more of the chronic complications, the major ones being neuropathy in 90(29.5%) nephropathy in 48 (15.7%) visual disturbance in 103 (33.8%) (*Table 2*).

**Table 2:** Distribution of chronic diabetic complications, JUSH diabetic follow-up clinic, October 2008.

	Frequency		
			Total
Chronic complication	Yes	No	
	No (%)	No (%)	
Visual disturbance	103(33.8)	202(66.2)	305
Neuropathy	90(29.5)	215(70.5)	305
Hypertension	76(24.9)	229(75.1)	305
Nephropathy	48(15.7)	257(84.3)	305
Skin infection	31(10.2)	274(89.8)	305
Foot ulcer/infection	14(4.6)	291(95.4)	305
Impotence	13(6.8)	179(93.2)	192

The occurrence of most complications (hypertension, visual disturbance, and neuropathy) increased with increasing age of the patient (P= 0.000). Type of DM was also significantly associated with all chronic complications (P= 0.000) with the exception of infection and foot ulcer; type II being more affected. The longer the duration of DM, the more frequent was the occurrence of the complications (P= 0.000). Large proportion (42.9%) of patients with diabetic retinopathy had diabetes for 5 to 9 years which was statistically significant (P = 0.001). Peripheral neuropathy was observed in 90 (29.5%) patients and of these 57 (63.3%) were type II diabetics (P = 0.000). Forty (44.4%) patients with peripheral neuropathy had diabetes for 5 to 9 years (P = 0.000). Diabetic foot ulcer was documented in 14 (4.5%) patients.

Ten (32.3%) of the patients with skin and subcutaneous tissue infection were from those who had diabetes for 5 to 9 years though (p value=0.000). Of the 31 (10.2%) patients who had dental problems, 18 (58.1%) were type II diabetics, 26(87.1%) had diabetes for less than 10 years.

Urine analysis and renal function test were documented only for 103 (33.7%) and 47 (15.4%) patients, respectively. Among these proteinuria was reported for 48 (45.7%) patients and eight (17.0%) had raised renal function test. Impotence was observed in 13 (6.8%) males and there was statistically significant association between impotence and diabetic age (P = 0.000) (*Table 3*).

**Table 3:** Chronic complications by sex, age, duration of illness and type of diabetes. Jimma University specialized Hospital, October 2008

Characteristics (N=305)	Had Complications						
	Hypertension (n=76) N (%)	Visual disturbance (n=103) N (%)	Impotence (n=13) N (%)	Neuropathy (n=90) N (%)	Foot Ulcer (n=14) N (%)	Infection (n=31) Number (%)	Proteinuria (n=48) Number (%)
Sex Male Female	47(24.5) 29(25.7)	60(31.2) 43(38.1)	13(6.8)	53(27.6) 37(32.7)	8(4.2%) 6(5.3%)	16(8.3%) 15(13.3%)	31(16.1%) 17(15.0%)
P-value	0.817	0.182		0.342	0.645	0.168	0.954
Age 15–34 35–44 45–54 ≥55	2(2.1) 5(9.3) 34(44.2) 35(43.8)	15(16.0) 14(25.9) 34(44.2) 40(50.0)	2(2.2) 3(5.6) 4(5.2) 4(5.1)	14(14.9) 13(24.1) 29(37.7) 34(42.5)	3(3.2%) 2(3.7%) 5(6.5%) 4(5.0%)	8(8.5%) 2(3.75%) 13(16.9%) 8(10.0%)	10(10.6%) 6(11.1%) 11(14.3%) 21(26.3%)
P-value	0.000	0.000	0.054	0.000	0.757	0.087	0.057
Type of DM Type I Type II Type II with 2^0^ failure	3(2.6%) 58 (37.9%) 15 (41.7%)	21 (18.1%) 67 (43.8%) 15 (41.7%)	3(2.6%) 8(5.3%) 2(5.6%)	19(16.4%) 57(37.3%) 14(38.9)	4(3.4%) 6(3.9%) 4 (11.1%)	9(7.8%) 13(8.5%) 9(25.0%)	11(9.5%) 25(16.3%) 12(33.3%)
P-value	0.000	0.000	0.003	0.000	0.642	0.371	0.018
Duration of illness < 5 5–9 10–14 15–19 >20	28 (17.2%) 29 (28.2%) 12 (42.9%) 3(50.0%) 4(80.0%)	28 (17.2%) 52 (50.5%) 17 (60.7%) 3(50.0%) 3(60.0%)	4(2.5%) 3(2.9%) 5(17.9%) 0(0%) 1(20%)	29(17.8%) 40(38.8%) 16(57.1%) 2(33.3%) 3(60%)	5(3.1%) 5(4.9%) 3 (10.7%) 0(0.0%) 1 (20.0%)	9(5.5%) 10(9.7%) 7(25%) 2(33.3%) 3(60%)	19(11.7%) 17(16.5%) 7(25.5%) 1(16.7%) 4(80.0%)
P-value	0.000	0.001	0.012	0.000	0.523	0.000	0.001

## Discussion

Among the 305 diabetic patients in this study, the majority were type II which is consistent with other reports in Ethiopia (3). DM was associated with hypertension in 76 (24.9%) patients which is higher than previous report from the same hospital ten year's back (7); the possible explanation for the discrepancy could be the fact that patients on follow up might have developed hypertension, as diabetic age increases the risk of developing hypertension

Diabetic ketoacidocis (DKA) occurs both in type I and type II DM with increased susceptibility in type I (5). Of the 66 patients who had DKA in this study, nearly three quarter were from type I and the rest were from type II diabetics. In other words more than a third (40/116) of the type I diabetics in this study had DKA, which is consistent with other report (8). The proportion of type II diabetics having DKA was also comparable to what had been reported (8), where about 22% of patients admitted with DKA were type II diabetics.

Among those who complained visual disturbances, a third had fundoscopic examination, which is suboptimal compared to the guidelines for ongoing medical care for patients with diabetes (1), which recommends at least annual examination for all diabetics. More than half of the studied cases had one or more of the chronic complications which is higher than the result from Menilik II hospital (4). The reason for this discrepancy needs further study to ascertain the differences.

Among those who underwent fundoscopic examination, 7 had diabetic non-proliferate stage retinopathy of which 5 had hypertension. Retinopathy was significantly associated with hypertension and diabetic age, which is similar to a report from Addis Ababa (5). The report from Addis Ababa indicated that the prevalence of diabetic retinopathy was comparable between type I and II diabetics. However, in this study the majority of patients with diabetic retinopathy were type II. This may be due to the facts that 29 out of the 33 patients, who had fundoscopic examination most were type II diabetics. Individuals with diabetic nephropathy commonly have diabetic retinopathy (1). Occurrence of diabetic nephropathy showed difference between type I and II but not statistically significant unlike a report from Menelik II hospital (4). The possible reason for the discrepancy could be that most patients were not evaluated for nephropathy in this study.

Among 192 male diabetic patients impotence was reported in only 13 (6.8%). This was much lower than two reports from Addis Ababa where they reported 55% and 48.7% prevalence, respectively (7, 9). Diabetic age and impotence were significantly associated which is in line with a study done in Addis Ababa (7). Unlike a report from Addis Ababa (10) the majority of men with impotence had type II diabetics which could be due to the few number of patients with documented impotence in this study.

One of the risk factors for the development of diabetic foot ulcer is having the diseases for more than 10 years (1). In contrary, more than two third of the patients with diabetic foot ulcer had diabetes for less than 10 years in this study. This difference could be due to underreporting, poor record keeping and delayed diabetic diagnosis.

Nearly half of the cases with neuropathy had the disease for five to nine years with statistically significant association. This is in contrast to reports from Tikur Anbessa and Menelik II hospitals (2, 10). The lower prevalence in this study could be due to under reporting and /or due to the fact that over three fourth of the studied subjects had the disease for less than ten years. One in ten patients had skin and subcutaneous tissue infection, one in twenty had diabetic foot ulcer and 8.5% had UTI. A study done in Tikur Anbessa Hospital reported skin and subcutaneous tissue infection in 12.8% of patients, diabetic foot infection in 35% and UTI in 14% (11). This discrepancy could be due to under reporting of cases in this study.

In conclusion, the majority of patients were type II diabetics, acute complications were observed more commonly among type I diabetics and DKA was the commonest acute complication. The frequency of chronic complications was high. Increased occurrence of retinopathy, peripheral neuropathy, impotence and nephropathy was observed with longer duration of illness. Impotence and diabetic nephropathy were more common in type II diabetics. The study also showed that recommended follow-up examinations and laboratory tests are neither done nor documented for large proportion of patients. Clinicians working in the clinic are advised to carry out necessary examinations and tests and make proper recordings.
